# Size Selective Harvesting Does Not Result in Reproductive Isolation among Experimental Lines of Zebrafish, *Danio rerio*: Implications for Managing Harvest-Induced Evolution

**DOI:** 10.3390/biology10020113

**Published:** 2021-02-04

**Authors:** Tamal Roy, Kim Fromm, Valerio Sbragaglia, David Bierbach, Robert Arlinghaus

**Affiliations:** 1 Department of Biology and Ecology of Fishes, Leibniz Institute of Freshwater Ecology and Inland Fisheries, Müggelseedamm 310, 12587 Berlin, Germany; kimfromm1990@hotmail.de (K.F.); bierbach@igb-berlin.de (D.B.); Arlinghaus@igb-berlin.de (R.A.); 2 Department of Marine Renewable Resources, Institute of Marine Sciences (ICM-CSIC), Passeig Marítim de la Barceloneta, 37-49, E-08003 Barcelona, Spain; valeriosbra@gmail.com; 3 Division of Biology and Ecology of Fishes, Department of Crop and Animal Sciences, Faculty of Life Sciences, Humboldt-Universität zu Berlin, 10117 Berlin, Germany; 4 Division of Integrative Fisheries Management, Department of Crop and Animal Sciences, Faculty of Life Sciences, Humboldt-Universität zu Berlin, 10115 Berlin, Germany

**Keywords:** fisheries-induced evolution, body size, prezygotic preference, reproductive allocation, zebrafish

## Abstract

**Simple Summary:**

Mortality in fish populations is commonly size-selective. In fisheries, larger fish are preferentially caught while natural predators preferentially consume smaller fish. Removal of certain sized fish from populations and elevated fishing mortality constitute a selection pressure which may change life-history, behaviour and reduce adult body-size. Because behaviour and body-size are related and influence mating preferences and reproductive output, size-selective mortality may favour subpopulations that less readily mate with each other. Our aim is to test this possibility using three experimental lines of zebrafish (Danio rerio) generated in laboratory by removing large-sized, small-sized and random-sized fish for five generations. We tested mating preferences among males and females and tested if they spawned together. We found males and females of all subpopulations to reproduce among themselves. Females generally preferred large-sized males. Females of all lines spawned with males, and males of all lines fertilised eggs of females independent of the subpopulation origin. Our study shows that size-selective mortality typical of fisheries or in populations facing heavy predation does not result in evolution of reproductive barriers. Thus, when populations adapted to fishing pressure come in contact with populations unexposed to such pressures, interbreeding may happen thereby helping exploited populations recover from harvest-induced evolution.

**Abstract:**

Size-selective mortality is common in fish stocks. Positive size-selection happens in fisheries where larger size classes are preferentially targeted while gape-limited natural predation may cause negative size-selection for smaller size classes. As body size and correlated behavioural traits are sexually selected, harvest-induced trait changes may promote prezygotic reproductive barriers among selection lines experiencing differential size-selective mortality. To investigate this, we used three experimental lines of zebrafish (*Danio rerio*) exposed to positive (large-harvested), negative (small-harvested) and random (control line) size-selective mortality for five generations. We tested prezygotic preferences through choice tests and spawning trials. In the preference tests without controlling for body size, we found that females of all lines preferred males of the generally larger small-harvested line. When the body size of stimulus fish was statistically controlled, this preference disappeared and a weak evidence of line-assortative preference emerged, but only among large-harvested line fish. In subsequent spawning trials, we did not find evidence for line-assortative reproductive allocation in any of the lines. Our study suggests that size-selection due to fisheries or natural predation does not result in reproductive isolation. Gene flow between wild-populations and populations adapted to size-selected mortality may happen during secondary contact which can speed up trait recovery.

## 1. Introduction

Fisheries constitute a global example of human-induced environmental change, which has fostered adaptive changes in a range of traits in exploited fish populations [1,2]. Intensive and trait-selective exploitation can induce evolutionary and other adaptations in life history, behaviour and physiological traits [2,3,4]. Most fishing gears are positively size-selective. In turn, larger sized individuals are particularly targeted in both commercial and recreational fisheries [5]. However, some fishing gears, such as gill nets, show dome-shaped selectivity where fish of intermediate size are preferentially harvested, and specific fishing regulations, such as maximum size limits, may focus on exploitation of smaller size classes while saving the larger ones [6]. Moreover, most fish populations are exposed to gape limited predation, which can result in negative size selection by preferentially harvesting the smallest size classes [7,8]. Both positive and negative size-selection can demographically and evolutionarily alter the size distribution in exploited fish stocks [9]. Because body size plays an important role in mate choice and sexual selection [10,11], harvest-induced adaptations in body size may alter mate choice patterns in exploited populations, which in turn can affect reproductive output [12,13,14]. In this study, we investigate if size-selective mortality affects sexual selection [15] and ask whether this may lead to reproductive barriers among exploited populations [16].

Sexually selected traits, like body size and certain behavioural phenotypes (e.g., boldness), are under direct and indirect influences of size-selective mortality [2,15,17]. Removing larger individuals from populations over successive generations can foster the evolution of a fast life history [2,18,19] characterised by fast juvenile growth rate, early sexual maturation at small size, increased reproductive investment and reduced post maturation growth [8,19,20,21]. Life history, morphology and behavioural traits are often correlated (following the pace-of-life hypothesis: [22]). Therefore, behavioural types (sometimes referred to as personality traits, defined as consistent among individual differences in behaviour: [23]), such as boldness, aggressiveness and activity [20,24,25], may coevolve with a fast life history in response to intensive and size-selective fisheries.

In fish species where body size forms the basis of mate choice, reductions in average body size due to harvesting could have an impact on mating systems [15]. In many species, large body size is an indicator of good genes or generally higher fitness [26,27], and males and females in many fish species tend to prefer large sized individuals as mating partners [28,29,30]. Females of certain fish prefer large and dominant males because they produce more sperm (mandarinfish *Synchiropus splendidus*: [31], mosquitofish *Gambusia holbrooki*: [32]), promising higher reproductive success, or because larger males defend territories and offspring more effectively (threespine sticklebacks *Gasterosteus aculeatus*: [33], Atlantic molly *Poecilia mexicana*: [34]). Consequently, in some fish species females have been found to allocate more reproductive resources to large males Banggai cardinal fish *Pterapogon kauderni*: [35], chinook salmon *Oncorhynchus tshawytscha*: [36], swordtail fish *Xiphophorus multilineatus*: [37]). However, under certain situations females may avoid highly aggressive males, often the largest member of a cohort, as they might lower their reproductive success due to sexual harassment [38,39]. Males also often prefer to spawn with larger females. This is because large females are more fecund and produce more eggs or offspring (guppy *Poecilia reticulata*: [40], two-spotted goby *Gobiusculus flavescens*: [41], annual fish *Austrolebias reicherti*: [42], Atlantic mollies *Poecilia mexicana*: [43], pink salmon *Oncorhynchus gorbuscha*: [44], Atlantic cod *Gadus morhua*: [45]). Because both males and females tend to prefer larger individuals, fisheries-induced evolution of small body size and associated changes in behaviour in the exploited populations may alter mate choices [14,16,46,47,48]. In particular, fisheries might promote small, and generally shy and less aggressive individuals [49], which are not necessarily the preferred mating partners, and in turn may strongly affect sexual selection patterns [15].

Smaller individual body size in exploited fish populations may affect sexual selection by altering choosiness for mates [15]. Specifically, the reduction in the number of preferred mates due to removal of larger individuals from populations is suggested to increase the proportion of non-preferred mates [50,51], and this can cause shifts in preference towards the latter when compared with natural, unexploited populations [15]. For example, in European lobsters *Homarus gammarus*, positive size-selective harvesting of males with relatively large claws resulted in changes in female preferences towards smaller mating partners with smaller claws, which lowered the reproductive output [48,52]. Generally, harvest-induced changes in mating systems (like mate choice, intrasexual competition and dominance) can change the speed, extent and direction of the evolving traits [14,53]. Change in mating preferences among exploited populations relative to unexploited ones may in turn foster assortative mating among subpopulations, and development of premating assortative preferences and mating barriers may result in reproductive isolation in the long term (e.g., cichlids: [54]). Reproductive isolation could, in turn, prevent trait and population recovery [16] even when subpopulations adapted to fishing pressure come into secondary contact with conspecifics residing in protected sites [13,55]. In extreme cases of reproductive isolation, speciation may occur because there will be limited or no gene flow among populations adapted to fisheries or other forms of size-selection [56,57,58]. Relatedly, the speed of fisheries-induced evolution might increase as the small-bodied individuals become less preferred mating partners among natural stock components thereby further increasing the selection pressures favouring small body size [14]. Despite this being an interesting hypothesis, there is lack of empirical evidence for assortative mating and reproductive isolation among exploited populations in response to size selection, (but see studies in European lobsters and zebrafish that first addressed this issue [16,53]).

We used experimental selection lines of zebrafish (*Danio rerio*) subjected to positive (Large-harvested, LH), negative (Small-harvested, SH) and random (Control, RH) size-selective mortality for five consecutive generations [19] and tested the potential for prezygotic reproductive isolation based on line-assortative association preferences. Previous studies with these experimental lines have shown that the three lines differ in body size, reproductive effort [19] and composition of behavioural phenotypes [19,59,60,61]. The selection lines significantly differed in broad scale gene expression and Single Nucleotide Polymorphism (SNP) allele frequencies [19,61,62], while the global genetic diversity among generations at the onset and after five generation of selection was maintained (Uusi-Heikkilä et al., unpublished data). The large-harvested line mimics populations in most exploited fisheries where the larger individuals are harvested. This line developed a fast life history and reached a smaller terminal body size and weight, matured earlier and had higher relative fecundity relative to the control [19]. The small-harvested line represents natural populations where the smallest size classes are exposed to gape-limited predation or populations in fisheries where maximum-size limits exist. This line is characterized by reduced reproductive allocation but it reaches a similar terminal body size compared to the control line, suggesting an evolution towards a slow life history [19]. Both small and large-harvested lines evolved altered maturation schedules by maturing at a smaller size and younger age compared to the control line fish [19]. The small body size of the large-harvested line fish [19] could make them less attractive to other populations as mating partners, which in turn may manifest into line-assortative mating and reproductive isolation. Yet, an experimental study by Sbragaglia et al. [16] did not find evidence for line-assortative mating among the zebrafish selection lines. Sbragaglia et al. [16] controlled for body size in their full factorial spawning experiments, i.e., all possible differences among selection lines were due to differences in behaviour or external features like coloration patterns. Further investigation of the impact of size differences among lines on the reproductive output is important, as the lines significantly differ in adult body size [19]. Size greatly influences mating behaviour [38,39,63,64], social preferences [65] and personality [66,67,68] in zebrafish. Larger zebrafish are bolder [depending on the native environment, 67]) and more active than smaller individuals [66], and large zebrafish may thus have higher reproductive success [64]. In previous studies, large male zebrafish have been shown to receive more eggs from females through differential allocation [39,63], though this result was not always found [69]. Female zebrafish are known to prefer larger and dominant mating partners [65,70], and dominant individuals have been found to be more successful in competitive exclusion of subordinate males resulting in higher reproductive success [71,72]. Therefore, the lack of evidence for reproductive isolation among the three zebrafish selection lines reported by Sbragaglia et al. [16] may not hold when the lines vary by size. The large-harvested line, which is significantly smaller than the other two lines [19], might then no longer be a preferred mating partner.

Here we test if size-selective harvesting has led to prezygotic reproductive barriers among the selection lines of zebrafish. We tested association preferences of males and females through dichotomous choice tests, and assessed reproductive allocation through subsequent spawning trials as in Sbragaglia et al. [16] but without controlling body size variation among the three lines. To test if differences in body size among selection lines led to prezygotic reproductive barriers, we compared results of statistical models constructed to analyse association preferences with and without individual body size as covariate. We expected that focal males will spend more time in association with larger females and focal females will spend more time in association with larger males, independent of the selection lines. For testing reproductive success, we conducted group spawning trials among selection lines in full factorial designs using two females and four males. We expected larger females to spawn more eggs and larger males to receive and fertilise more eggs independent of the selection lines, and expected to find no evidence for line-assortative reproductive output in line with previous studies [16].

## 2. Material and Methods

### 2.1. Selection Lines

We used F_13_ of the three selection lines of zebrafish (small, large and random-harvested lines, each with a replicate, i.e., six lines in total) described in [19]. These lines were produced through directional size-selection (i.e., 75% per generation harvest rate) applied to wild caught fish over five generations (F_1_ to F_5_), and cessation of selection after that for several generations to remove maternal effects [73]. In the small-harvested line, 25% of the largest individuals were used as parents in the successive generations while in the large-harvested line, 25% of the smallest individuals were used as parents in successive generations. In a separate control group, 25% of random individuals were selected for reproduction every generation. Timing of harvest depended on when half of the control line became mature [19]. The selection lines differed in body size and life history [19] as described previously and these evolved differences were maintained after size-selective harvesting was stopped, as confirmed by Sbragaglia et al. [16,61] at F_9_ and F_13_ (Supplementary Figure S1). Previous work revealed that the selection lines differed in broad scale gene expression [61,62], and that the phenotypic differences had a genetic underpinning [19] and were not just results of plasticity.

**Figure 1:**
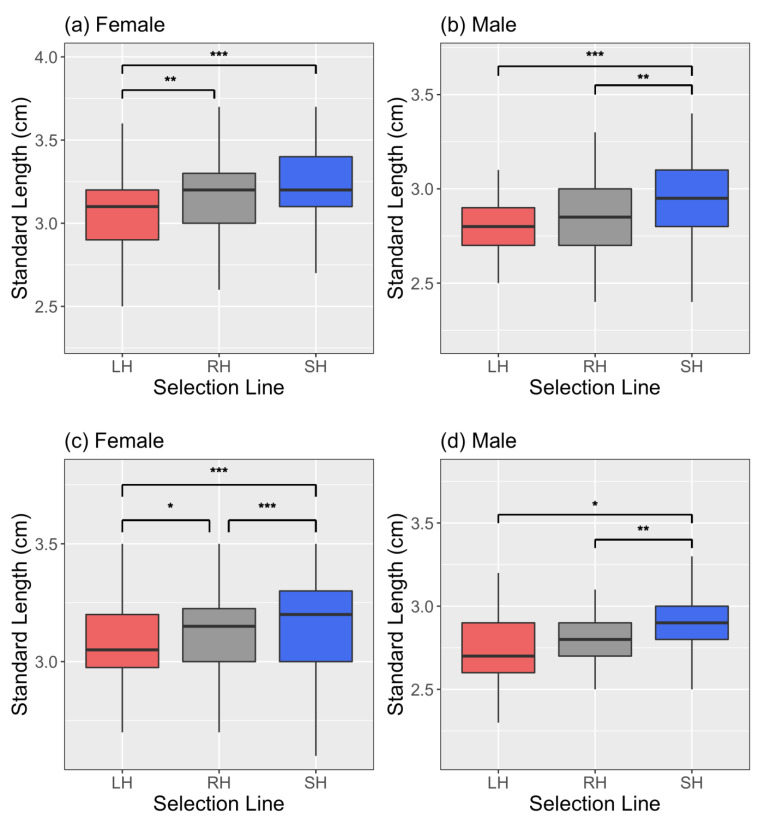
Comparison of body size of individuals across selection lines (Large-harvested (LH), Random-harvested (RH) and Small-harvested (SH)) among (**a**) females and (**b**) males in prezygotic preference tests, and (**c**) females and (**d**) males in spawning trials. Significant results are indicated by *. Significance codes: (*p* ≤ 0.001) ‘***’, (*p* ≤ 0.001) ‘**’, (*p* ≤ 0.01) ‘*’.

In this study, the F_13_ fish from the three selection lines continued to significantly differ in body size as shown by Sbragaglia et al. [61]. The small-harvested line fish were significantly larger, and the large-harvested line fish significantly smaller than the control line in the adult life stage (Figure 1, Supplementary Table S1). Specifically, random-harvested line females were significantly larger than large-harvested line females (Figure 1a), while small-harvested line males were significantly larger than random-harvested line males in prezygotic trials (Figure 1b). In the spawning trials, small-harvested line females were significantly larger than random-harvested line females, which were larger than large-harvested line females (Figure 1c). Here, small-harvested line males were significantly larger than random-harvested line males (Figure 1d).

We housed fish from the selection lines in the laboratory in six round holding tanks (diameter: 79 cm, height: 135 cm, volume: 320 L) at a density of 1300 per tank and a 14:10 h light:dark cycle. Water in the tanks was maintained at a temperature of 27 °C, pH 8.5 by a circulation system, and the fish were fed with commercial flake food (TetraMin Tropical).

### 2.2. Prezygotic Tests

**Figure 2:**
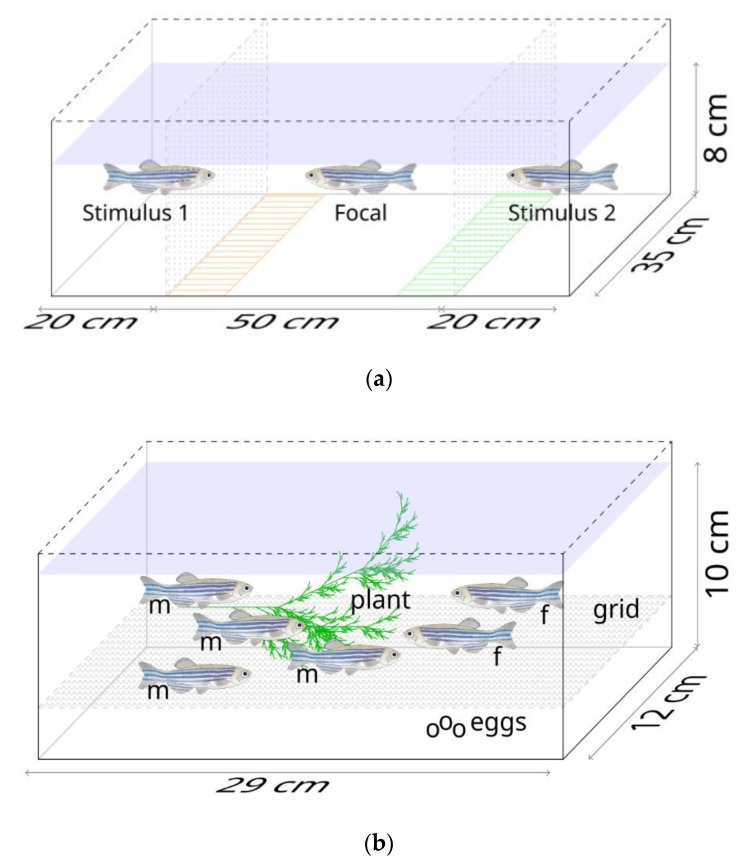
Diagrammatic representation of experimental setups for (**a**) prezygotic preference tests and (**b**) spawning trials. In the prezygotic setup, the orange and green areas represent preference zones. The postzygotic setup held two females “f” and four males “m”. The image of zebrafish used has been taken from the source https://commons.m.wikimedia.org/wiki/File:201108_zebrafish.png, accessed on 20 August 2020 (author: DataBase Center for Life Science (DBCLS)), licensed under Creative Commons Attribution 4.0 International license (https://creativecommons.org/licenses/by/4.0/deed.en, accessed on 16 October 2020).

We tested preferences of individual (focal) fish for two stimulus fish, one from the same and one from a different selection line. Dichotomous choice tests have been used intensively to test premating preferences in fish [74,75]. We used a rectangular glass tank (90 cm × 35 cm × 35 cm) and divided it lengthwise into three compartments; a central (50 cm × 35 cm × 35 cm) and two flanking compartments (20 cm × 35 cm × 35 cm) by transparent permeable acrylic dividers (Figure 2a). We covered the outer sides of the tank with white sheet so that the fish were not distracted by external disturbances.

We used a total of 720 fish from the selection lines. We used 240 individuals as focal fish (120 males and 120 females) and 480 individuals as stimulus fish (240 males and 240 females) for the experiments, selecting 40 individuals per line as focal fish and 80 individuals per line as stimulus fish. We used six possible combinations of selection lines for the focal and the stimulus fish, and we randomised the order in which these combinations were tested. Before the experiments, we randomly selected fish from the holding tanks and sorted them based on sex. We conducted the experiments between 11 and 16 h, and tested 12 combinations of focal and stimulus fish every day. We filled the experimental tank with 25 L of water to a level of 8 cm. We then transferred a single individual (focal fish) into the central compartment of the tank. We randomly selected two stimulus fish of the opposite sex (male stimuli in case of a female focal fish, and vice versa), one from the same selection line as the focal fish and one from a different line (e.g., large-harvested focal females paired with large- and small-harvested stimulus males) and placed them into the two flanking compartments. This resulted in two combinations of stimulus fish for focal fish of each line, therefore six combinations in total. The fish were allowed to acclimate for one min and we then recorded the behaviour of the focal individual for five min using an overhead webcam (Logitech B910). To avoid side biases, we swapped positions of the two stimulus fish and repeated the experiment immediately after the first trial. From the video recordings, we scored the time spent by the focal fish near the two stimulus fish (association time) in the preference zones (Figure 2a) in a fully blinded way using EthoVision XT 9 (Noldus IT, Wageningen, The Netherlands), and combined the measures from the consecutive experiments where positions of the stimulus fish were changed. We measured the standard length of the focal and stimulus fish at the end of the experiment. The fish were transferred to a separate holding and were not used again. All fish were fed normally after the experiments.

### 2.3. Spawning Trials and Reproductive Output

We examined reproductive success (egg allocation and percentage of eggs fertilised) across selection lines by conducting group spawning trials following the protocol of our laboratory [16,19]. We used a full factorial design and a random set of fish from each of the selection lines, thereby not controlling for body size. By full factorial design we meant that males and females of each selection line mated with females and males of all the lines. This meant that large-harvested females mated with males of all the lines (large-harvested × large-harvested, large-harvested × random-harvested, large-harvested × small-harvested) and similarly did random-harvested and small-harvested females, giving rise to nine possible mating combinations. We used rectangular acrylic reproduction boxes (29 cm × 12 cm × 10 cm) with a grid fitted 3 cm above the bottom of the box that allowed eggs to fall through, and a green plastic mesh that mimicked a plant (Figure 2b). A total of 540 individuals (360 males and 180 females) were used in the spawning trials. We conducted 10 replicates for each combination over a period of 10 weeks. For spawning, we selected two females and four males randomly from the stock, transferred them into the breeding box and allowed them to breed over four consecutive days following previously established protocols of our lab [64]. Zebrafish reproductive behaviour is mostly restricted to the early morning hours [76,77]. Therefore, we collected eggs every morning after first two hours of artificial daybreak (lights on) and counted the number of fertilised and unfertilised eggs under a binocular stereomicroscope (Olympus SZ series). We fed the fish with live *Artemia* once daily. At the end of the four-day spawning period, the fish were transferred to a separate holding and were not used again.

### 2.4. Statistical Analysis

We constructed separate linear mixed effects regression models to test prezygotic preferences of male and female focal fish from the three selection lines. We first ran models without including body length as covariate and later added body length to the models to isolate the size effects. Thereby, we modelled the response variable with and without body size as a covariate to distinguish between line-specific differences that were not due to line differences in body size (e.g., due to behaviour, body colouration). We transformed the data using Tukey’s Ladders of Powers transformation and used the transformed variable to fit linear mixed effects models. We used “TukTime” (Tukey transformed measure of association time) as a dependent variable, interaction of “Stimulus” (selection line) and “StimulusType” (same or different than focal fish) as a fixed effect, and “Pair” (focal fish) and “StimulusID” (to account for selection line replicates) as random intercepts. To test if time spent by focal fish depended on body size of the stimulus fish, we constructed models using interaction of “Stimulus” (selection line), “StimulusSL” (standard length of the stimulus fish) and “StimulusType” (same or different than focal fish) as a fixed effect, and “Pair” (focal fish) and “StimulusID” (to account for selection line replicates) as random effects.

We constructed linear mixed effects regression models to test if the number of eggs spawned and percentage of eggs fertilised differed across different combinations of male and female selection lines in the tests for reproductive output. As before, we transformed the dependent variables using Tukey’s Ladders of Powers transformation. We used the total number of eggs as a dependent variable, interaction of “Male” (selection line of male fish) and “Female” (selection line of female fish) as fixed effect, and “MaleID” and “FemaleID” (selection line replicates) as random intercepts. We similarly ran mixed effects models to test differences in percentage fertilised eggs across different selection line combinations. As the experiments were conducted in groups, we did not include standard length of fish as a co-variate in the models.

All analyses were conducted in R version 3.6.1 [78]. Data were transformed using the “rcompanion” package [79] and analysed using the “lmerTest” package [80].

## 3. Results

**Figure 3:**
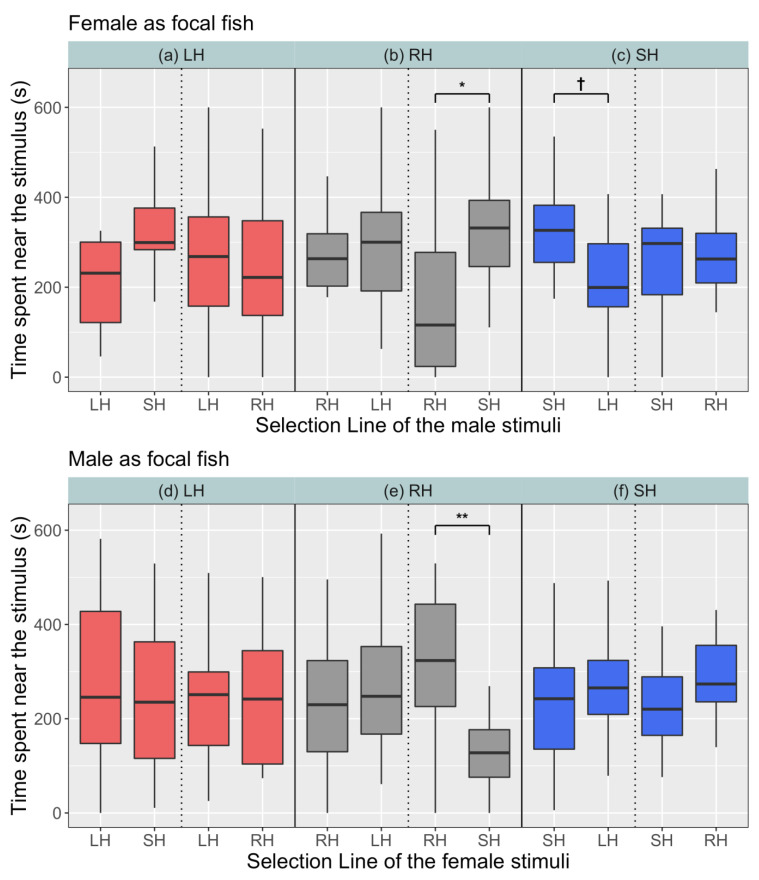
Comparison of prezygotic preferences of female ((**a**–**c**)), and male focal fish ((**d**–**f**)) across selection lines. The upper *x*-axis represents focal fish and the lower *x*-axis represents stimulus fish from the same or different selection lines. Significance codes: (*p* < 0.01) ‘**’, (*p* = 0.01) ‘*’, (*p* > 0.05) ‘†’.

In the prezygotic preference trials without including body size as a covariate, we observed assortative preferences only for control line males and not females. Control line females spent significantly more time in association with males of the small-harvested line (325 ± 33 s) than their own line (165 ± 37 s) (t = −2.5, *p* = 0.01) (Figure 3b), which implied that there was no evidence for line-specific preferences in control females. By contrast, the small-harvested line females spent more time in association with the males of their own line (311 ± 31 s) than with males of the large-harvested line (216 ± 31 s), but this was not statistically significant (t = 1.7, *p* = 0.08; Figure 3c). For males, we found that control line males spent significantly more time in association with females of their own line (325 ± 31 s) than with females of the small-harvested line (135 ± 21 s) (t = −2.9, *p* < 0.01; Figure 3e). Females and males of the other lines did not differ significantly in the time spent in association with males and females of self and foreign lines (Table 1, Figure 3).

**Figure 4:**
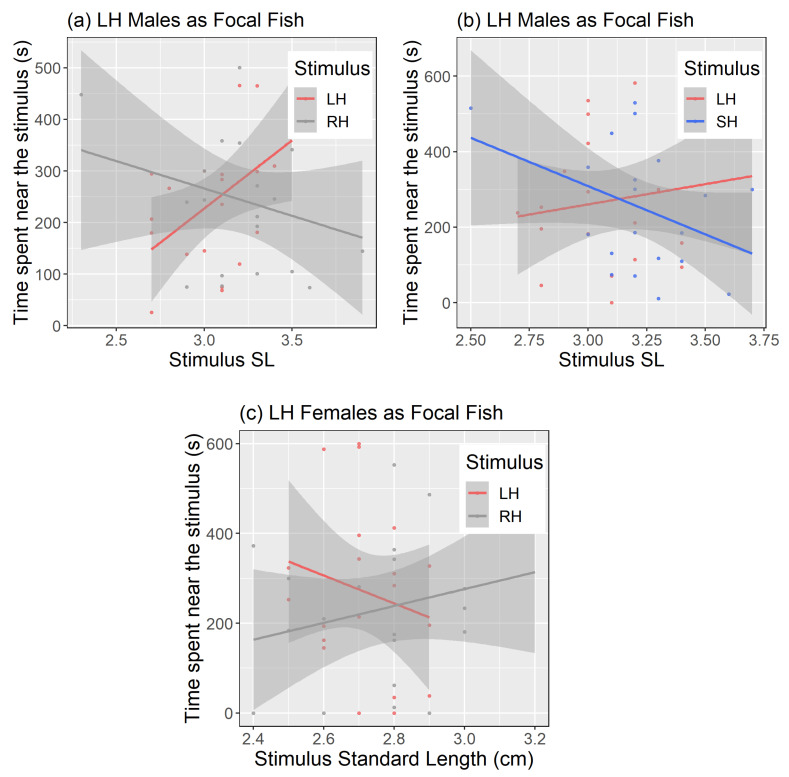
Interactions of body size (SL) of stimulus fish and time spent by LH male (**a**,**b**) and LH female (**c**) focal fish near stimulus fish. Shaded areas spanning the regression lines represent confidence intervals. SL = Standard length; LH = Large-harvested; RH = Random-harvested; SH = Small-harvested.

When body size was included as a covariate, relationships changed and significant interaction terms among the selection lines and body size were revealed (Table 1). Specifically, we found that large-harvested line males spent significantly more time in association with large females of their own line compared to similarly large females of random-harvested (t = −2, *p* < 0.05; Figure 4a) and small-harvested lines (t = −2.6, *p* = 0.01; Figure 4b). This finding suggested both line-assortative and size dependent preferences in large-harvested line males. In females, large-harvested line females spent more time in association with larger males of the random-harvested line (t = 2.0, *p* < 0.05; Figure 4c). No other association preferences were revealed (Table 1), and the patterns of line-assortative prezygotic preferences were thus, overall, very weak.

**Figure 5:**
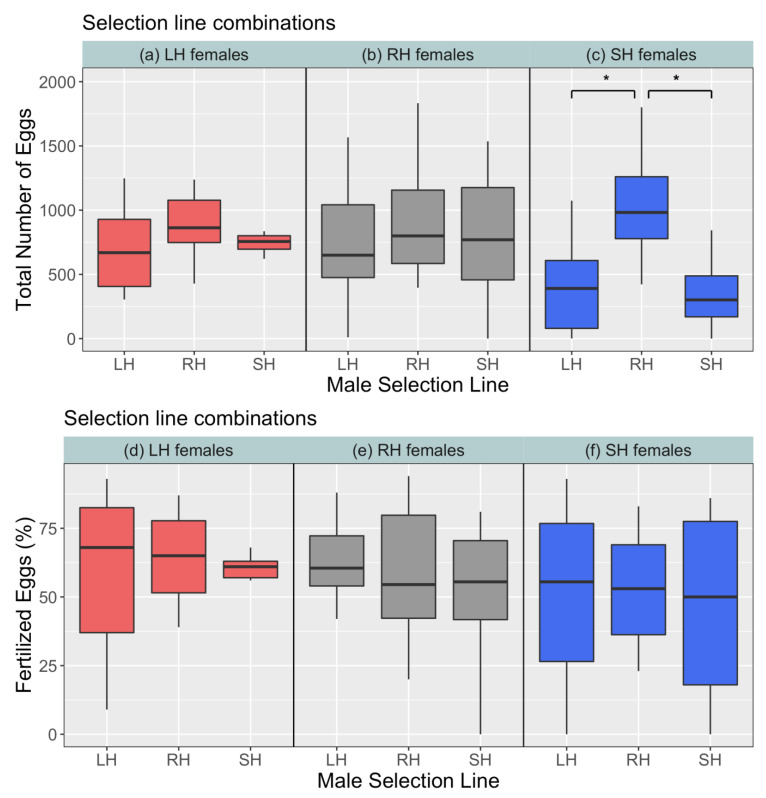
Comparison of total no. of eggs spawned by females (**a**–**c**) and percentage of eggs fertilised by males (**d**–**f**) among the selection lines. Significance code: (*p* < 0.05) ‘*’.

In the tests for reproductive output (spawning trials), we did not find any evidence of line-assortative reproductive allocation. The number of eggs spawned depended on the selection line of males (F_2,77_ = 9.3, *p* < 0.01), and not females (F_2,3_ = 0.4, *p* = 0.68; Table 2). Males of the random-harvested line received significantly more eggs (1074 ± 140) from females of the small-harvested line than males of other lines (LH males: 425 ± 120 eggs; SH males: 391 ± 102 eggs) (t = 2, *p* = 0.04; Figure 5c). We did not detect any significant differences in percentage fertilised eggs as a function of male (F_2,80_ = 0.5, *p* = 0.59) and female (F_2,3_ = 0.5, *p* = 0.65) identity (Figure 5d–f), and thus no pattern of line-assortative reproductive output was revealed.

## 4. Discussion

Our study in an experimental zebrafish model system showed that five generations of size-selective harvesting followed by eight generations during which harvesting was halted affected prezygotic association preferences and also modestly affected reproductive success. Yet, we found only weak evidence for line-specific association preferences and these were often due to intrinsic preferences towards large body size and not due to an intrinsic preference for a certain line. In tests of reproductive output and allocation, females and males of the different lines readily spawned with, and fertilised eggs from, males and females of the other lines. Importantly, we did not find evidence of line-assortative reproductive allocation even though we did not control for body size in our study and despite the large- and small-harvested lines differing significantly in body size relative to the control. Our results reinforce the earlier findings of Sbragaglia et al. [16] that size-selective harvesting did not generate selection lines of zebrafish that more readily reproduced with each other than with other lines. Therefore, under the conditions of experimental evolution in our zebrafish model, there was no evidence that strong size-selection fosters reproductive isolation among the various selection lines.

In the prezygotic tests, without statistically controlling for body size, we found that control (random-harvested) line females significantly preferred small-harvested line males (Figure 3b). Females of other lines also preferred to be associated with small-harvested line males (Figure 3a–c). Among the fish used in the prezygotic tests, we found that small-harvested line males were significantly larger than random-harvested line males (Figure 1a, Supplementary Table S1). Therefore, females seemed to prefer small-harvested line males because of their large body size. Large males have higher reproductive success in zebrafish [39,64] and previous studies have shown that female zebrafish prefer large sized males [65] and spawn more frequently with them producing bigger clutches [63]. Alternately, behavioural differences among lines could be the reason behind preference of females for small-harvested line males. Females typically prefer bolder males as sexual partners in different species [81,82,83], and these bolder fish are often large sized [81,84]. Indeed, small-harvested line zebrafish have been found to be bolder and more active than the fish from the other lines [61] and this difference in personality could have also contributed to females preferring males of the small-harvested line.

We further found that control line males preferred control line females over small-harvested line females independent of body size (Figure 3e), suggesting evidence for line-assortative preferences in the control fish. However, control line females did not show a similar trend and, thus, the argument of line-assortative preference in the control line is only partially supported. Assortative preferences have been observed in many fish species like cichlids [85,86], *Poecilia mexicana* [87], sticklebacks [88] and the guppy [89] where there is a correlation among specific phenotypes (like colour, body size, or personality) between mated partners [90]. In zebrafish, assortative preferences are based on coloration patterns and personality traits [91,92]. Our results showing control line males preferring females of their own line could thus be explained by differences among lines in colouration or behaviour. Alternatively, rather than active preferences, the control line males could have avoided the small-harvested line females in the choice tests (Figure 3e), and this could, in turn, have manifested in the apparent preference for females of their own line. A previous study in western mosquitofish *Gambusia affinis* has shown that males strongly prefer more active females [81]. Small-harvested line females have been shown to be less active and less sociable compared to control line females [59], and therefore, they might have been less attractive to the control line males in the association tests.

When we accounted for body size in our models, we found that prezygotic preferences of only the large-harvested line fish was influenced by stimulus body size, and no other line showed significant association preferences. Large-harvested line males preferred larger females of their own line over similarly large females of other two lines, as evidenced by significant interaction effects of selection lines and body length (Figure 4a). Large females signal high reproductive success because they spawn more eggs and spawn more frequently than smaller females [39,64]. This explains the inclination of large-harvested males for large sized females. However, the preference for large sized females of their own line over other lines indicates exclusive assortative choice. Size-dependent assortative pairing and mating is common in the animal kingdom [90] where males and females tend to associate with partners of the same size. On the other hand, large-harvested line females preferred larger males of the random-harvested line over similarly large males of their own line (Figure 4b). One reason behind this could be that the control line males are less aggressive than other males [16]. Females tend to avoid choosing overtly aggressive males as mating partners because they reduce their reproductive success [38,93,94]. However, individuals were not in physical contact with each other in our choice experiments; therefore, male aggression may not be the only factor. Females could also exert a preference for bolder males with higher risk-taking tendencies [81,95,96]. Previous studies with the zebrafish lines have indicated that control line fish are bolder than the large-harvested line fish [60,61], and this difference in personality could explain why the former were preferred. Large males have higher reproductive success [39,63] and are more often chosen as mating partners in zebrafish [65,70]. We propose this as the reason why larger males of the control line were preferred in our association tests. Overall, however, we did not see alteration of mate preferences for larger individuals due to size selection. Fish preferred mates either from the small-selected line with larger average body size, or preferred larger individuals from among other lines. This is in contrast with a previous study on European lobsters where positive size-selective harvesting of males with relatively large claws resulted in changes in female preferences towards smaller mating partners with smaller claws [48].

We did not find any evidence of line-assortative reproductive success among males and females of the selection lines when conducting spawning trials as a measure of reproduction output. This means that neither the females spawned more eggs with males of their own line than other lines, nor did the males fertilise more eggs of females of their own line than other lines. Differential allocation of reproductive resources has been demonstrated previously in zebrafish where females spawned more eggs with larger males when choosing among small and large mating partners [63,70], and bolder and more aggressive males have been found to fertilise more eggs than shy and less aggressive males [71]. We found that small-harvested line females spawned significantly more eggs with control line males than large- and small-harvested line males (Figure 5a) in group spawning events. This result is in agreement with Sbragaglia et al. [16] where the authors found that control line males received more eggs than males of small- and large-harvested lines, while controlling for body size differences among the zebrafish selection lines. Sbragaglia et al. [16] also found that control line males were significantly less aggressive than males of the small- and large-harvested lines in spawning experiments. Male harassment of females during courtship and mating can have fitness consequences for females by increasing female costs for resource acquisition and reproduction [93,97,98]. In zebrafish, more aggressive males may not be successful in courting females more than less aggressive males [77]. Female reproductive allocation may also be reduced in the presence of very large and aggressive males [16,38,39,99]. Here we had two females and four males in the breeding boxes for a period of four days and this may have resulted in elevated aggression among males that could deter females [99,100]. We speculate that control line males received more eggs from small-harvested line females probably because they were less aggressive compared to large- and small-harvested line males. Such differential allocation could also be the result of female preferences for some unmeasured traits (e.g., colouration: [92]). Unlike Sbragaglia et al. [16] where males of the large-harvested line fertilised a higher percentage of eggs, we did not find differences in percentage of eggs fertilised among males of the three selection lines (Figure 5d–f), indicating equal fitness in all selection lines and no evidence for line-assortative patterns of reproductive output.

Our results from the tests for reproductive allocation were different from the preferences observed in zebrafish in the prezygotic tests. For example, small-harvested line females spawned more eggs with control line males but did not show significant association preferences for the latter (Figure 3c). This could be because the prezygotic tests were conducted outside the time window during which the zebrafish are sexually active. Courtship and spawning in zebrafish usually happen during the first two hours after artificial daybreak (lights on) in the laboratory [76,77]. Association preferences tested outside this time window could instead be indicative of shoaling tendencies rather than sexual motivation. Another uncertainty of this study is that the control group cannot be compared to wild or unexploited populations because our control line was also subjected to harvest, albeit non-randomly with respect to body length. Comparing results with a control population that has been subjected to minimal harvest would be a suitable avenue for further research [101].

The three selection lines revealed significant divergent selection responses in a range of outlier single nucleotide polymorphisms at F_6_ [19] and showed line-specific patterns in the transcriptomic profiles of the liver at F_11_ [62] and the molecular control of the circadian system at F_13_ [61]. This is evidence that five generations of selection left a genetic legacy in our zebrafish population [19], while maintaining basic genetic variability (Uusi-Heikkilä et al., unpublished data). Moreover, recent studies with these lines (using F_13_) showed differences in behavioural traits related to biological rhythms, feeding and mating [16,59,60]. Associated with the substantial molecular and phenotypic differences among the size-selected lines, we expected the evolution of line-assortative mating preferences and reproductive success. Our results, however, demonstrated a mating bias for large body-size in all three selection lines and no evidence for line-assortative reproductive allocation. These results can be argued as a form of evolutionary resistance in sexual selection for large body size [102]. A related evolutionary experiment where large-sized medaka *Oryzias latipes* were harvested over six generations also showed that some adaptive traits, such as maturation timing and somatic growth, did not respond to intensive anthropogenic selection [103]. Thus, some traits may simply not respond easily to anthropogenic selection [102]. In our study with zebrafish, the strong fitness benefits of large body-size in nature likely precluded the alteration of mating preferences for larger partners due to size-selection alone. Hence, even though the size-selected lines of zebrafish differ genetically and phenotypically in a range of traits, we did not see an associated change in mating preferences after five generations of intensive selection, or that any change in size-related mating preferences might have rebuilt during eight generations where no further selection acted on the fish.

## 5. Conclusions and Implications

Fisheries-induced evolution due to intensive size-selective harvesting can delay trait and population recovery [13,104,105]. One of the ways in which population recovery might not happen following secondary contact (with unexploited populations like in marine protected areas; [52]) is when there are evolved reproductive barriers between exploited and unexploited populations. Our study using an experimental system in the laboratory shows that highly intensive size-selective mortality at the rate of 75% per generation over five generations may not be strong enough to foster evolution of reproductive barriers among size-selection lines, even when they significantly differ in body length. Our work also implies that a strong selection on small body size may not be sufficient to cause reproductive isolation. We observed weak and inconsistent evidence for line-assortative preferences, and these were often influenced by body size independent of the selection line identity. Preference for mates from selection line with larger average body size, and preference for larger individuals within populations indicate that our size selection did not alter intrinsic mate preferences for larger bodied individuals, or any changes have already been reversed through counter selection after our harvest selection has stopped. We thus did not find support for line-assortative reproductive success even when lines differed strongly in mean body size. These results suggest that continued gene flow from wild or unexploited populations to populations evolutionarily adapted to high fishing pressure is conceivable, and perhaps very likely, and this may help in trait recovery once fishing ceases in highly exploited areas. Our laboratory study should only be carefully extrapolated to the wild and more studies in the wild are needed to test the validity of our experimental findings.

## Figures and Tables

**Table 1:** Results of fixed effects from linear mixed effects models with and without inclusion of body size to test differences in time spent by focal (a) female and (b) male fish near the stimuli. Significant results are in bold. SL = Standard length; LH = Large-harvested; RH = Random-harvested; SH = Small-harvested.

(a).							
Focal Line	Model	Term	Sum Sq.	Mean Sq.	df	F	Pr (>F)
LH	Without body size	Stimulus	44,162	22,081	2,2	1.87	0.34
	With body size	Stimulus	49,495	24,747.6	2,73	2.19	0.12
		StimulusSL	1249	1249.2	1,72	0.11	0.74
		Stimulus × Stimulus SL	51,239	25,619.3	2,73	2.27	0.11
RH	Without body size	Stimulus	127,492	63,746	2,77	5.07	**<0.01**
	With body size	Stimulus	29,216.4	14,608.2	2,74	1.14	0.32
		StimulusSL	261.6	261.6	1,74	0.02	0.89
		Stimulus × Stimulus SL	22,587.2	11,293.6	2,74	0.88	0.42
SH	Without body size	Stimulus	32,299	16,150	2,77	1.54	0.22
	With body size	Stimulus	8022.6	4011.3	2,74	0.37	0.69
		StimulusSL	3521.9	3521.9	1,74	0.33	0.57
		Stimulus × Stimulus SL	9232.4	4616.2	2,74	0.43	0.64
**(b).**							
**Focal line**	**Model**	**Term**	**Sum Sq.**	**Mean Sq.**	**df**	**F**	**Pr (>F)**
LH	Without body size	Stimulus	143.39	71.69	2,4	0.04	0.95
	With body size	Stimulus	12,475.9	6238	2,74	3.92	**0.02**
		StimulusSL	486.3	486.3	1,74	0.31	0.58
		Stimulus × Stimulus SL	12,731.7	6365.8	2,74	4.0	**0.02**
RH	Without body size	Stimulus	21,107	10,554	2,77	7.06	**<0.01**
	With body size	Stimulus	1097.8	548.89	2,74	0.36	0.70
		StimulusSL	1122.7	1122.69	1,74	0.74	0.39
		Stimulus × Stimulus SL	1284.3	624.17	2,74	0.41	0.66
SH	Without body size	Stimulus	4126	2063	2,5	2.32	0.19
	With body size	Stimulus	1978.12	989.06	2,74	1.1	0.34
		StimulusSL	661.42	661.42	1,74	0.73	0.4
		Stimulus × Stimulus SL	1565.05	782.52	2,74	0.87	0.42

**Table 2:** Results of fixed effects from linear mixed effects models to test differences in total no. of eggs spawned and percentage of eggs fertilised among different combinations of male and female selections lines. Significant results are in bold.

Metric	Term	Sum Sq.	Mean Sq.	df	F	Pr (>F)
	Male	117,875	58,938	2,77	9.34	**<0.01**
**Total no. of eggs**	Female	5493	2746	2,3	0.43	0.68
	Male × Female	52,307	13,077	4,77	2.07	0.09
	Male	35,061	17,530.4	2,77	0.57	0.57
**% fertilised eggs**	Female	20,845	10,422.3	2,3	0.34	0.73
	Male × Female	6777	1694.2	4,77	0.05	0.99

## Data Availability

The data used in this study are available as Supplementary Material.

## Supplementary Materials

The following are available online at https://www.mdpi.com/2079-7737/10/2/113/s1, Figure S1: Lester biphasic growth model, Table S1: Results of ANOVA comparing body size among selection lines.
