# Supplementary material for: Size Selective Harvesting Does Not Result in Reproductive Isolation among Experimental Lines of Zebrafish, Danio rerio: Implications for Managing Harvest-Induced Evolution
Source: Biology (Basel). 2021 Feb 4;10(2):113. doi: 10.3390/biology10020113 (PMC7913724; doi:10.3390/biology10020113)
Supplement: Supplementary file 1 [file biology-10-00113-s001.zip › Roy&FrommEt.al.2020SupplementaryMaterial.docx]

**Supplementary figures**


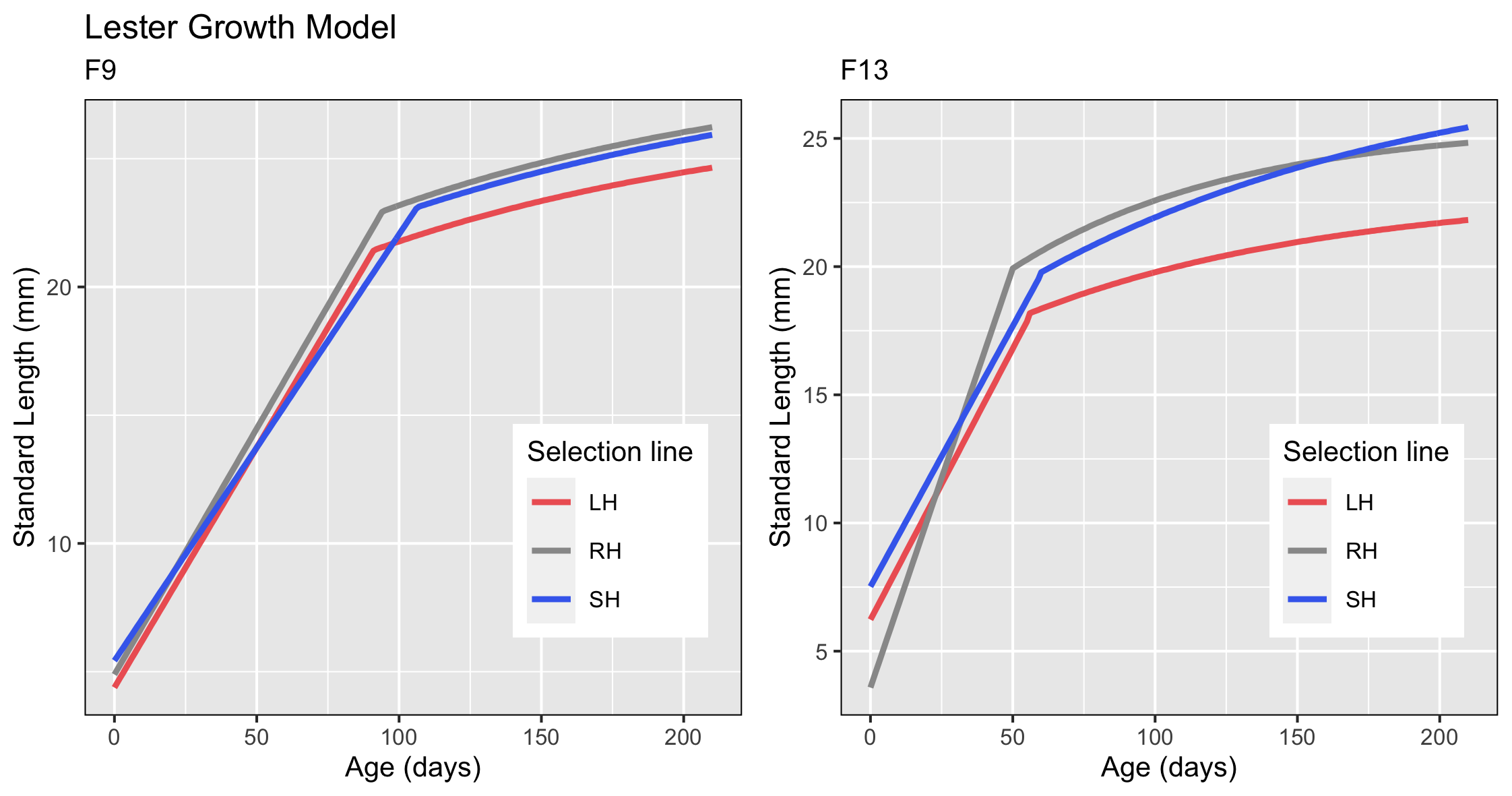


**Figure S1**: Lester biphasic growth model showing changes in body size with the age of fish at F9 and F13 generations.

**Supplementary Tables**

| **Sex** | **ANOVA** | **Df** | **Sum Sq.** | **Mean Sq.** | **F** | | **Pr(<F)** |
| --- | --- | --- | --- | --- | --- | --- | --- |
| **a)** | | | | | | | |
| Female | Selection line | 2, 357 | 1.97 | 0.98 | 14.98 | **<0.01** | |
| Male | Selection line | 2, 357 | 0.75 | 0.37 | 11.03 | **<0.01** | |
| **b)** | | | | | | | |
| Female | Selection line | 2, 535 | 1.19 | 0.94 | 16.68 | **<0.01** | |
| Male | Selection line | 2, 715 | 0.51 | 0.25 | 6.75 | **<0.01** | |

| **Sex** | **Contrast** | **Estimate** | **SE** | **Df** | **t.ratio** | **p** |
| --- | --- | --- | --- | --- | --- | --- |
| **c)** | | | | | | |
| Female | LH – RH | -0.11 | 0.033 | 357 | -3.25 | **<0.01** |
|  | LH - SH | -0.18 | 0.033 | 357 | -5.44 | **<0.01** |
|  | RH – SH | -0.07 | 0.033 | 357 | -2.19 | 0.07 |
| Male | LH – RH | -0.02 | 0.024 | 357 | -0.7 | 0.76 |
|  | LH – SH | -0.1 | 0.024 | 357 | -4.37 | **<0.01** |
|  | RH – SH | -0.087 | 0.024 | 357 | -3.67 | **<0.01** |
| **d)** | | | | | | |
| Female | LH - RH | -0.084 | 0.025 | 535 | -3.34 | **<0.01** |
|  | LH - SH | -0.144 | 0.025 | 535 | -5.75 | **<0.01** |
|  | RH - SH | -0.06 | 0.025 | 535 | -2.41 | **<0.05** |
| Male | LH - RH | 0.011 | 0.018 | 715 | 0.62 | 0.81 |
|  | LH - SH | -0.05 | 0.018 | 715 | -2.83 | **<0.05** |
|  | RH - SH | -0.061 | 0.018 | 715 | -3.45 | **<0.01** |

**Table S1:** Results of ANOVA comparing body size among females and males of the selection lines in a) prezygotic preference tests and b) spawning trials, and Posthoc Tukey tests to detect differences among lines for c) prezygotic tests and d) spawning trials. Significant results are in bold.
